# Effects of Cholesterol on Water Permittivity of Biomimetic Ion Pair Amphiphile Bilayers: Interplay between Membrane Bending and Molecular Packing

**DOI:** 10.3390/ijms20133252

**Published:** 2019-07-02

**Authors:** Wu-jhao Tien, Kun-you Chen, Fong-yin Huang, Chi-cheng Chiu

**Affiliations:** 1 Department of Chemical Engineering, National Cheng Kung University, Tainan 70101, Taiwan; 2 Hierarchical Green-Energy Materials (Hi-GEM) Research Center, National Cheng Kung University, Tainan 70101, Taiwan

**Keywords:** biomimetic membrane, ion pair amphiphile, cholesterol, molecular dynamics, water permeation

## Abstract

Ion pair amphiphile (IPA), a molecular complex composed of a pair of cationic and anionic amphiphiles, is an inexpensive phospholipid substitute to fabricate vesicles with various pharmaceutical applications. Modulating the physicochemical and permeation properties of IPA vesicles are important for carrier designs. Here, we applied molecular dynamics simulations to examine the cholesterol effects on the structures, mechanics, and water permittivity of hexadecyltrimethylammonium-dodecylsulfate (HTMA-DS) and dodecyltrimethylammonium- hexadecylsulfate (DTMA-HS) IPA bilayers. Structural and mechanical analyses indicate that both IPA systems are in gel phase at 298 K. Adding cholesterol induces alkyl chain ordering around the rigid sterol ring and increases the cavity density within the hydrophilic region of both IPA bilayers. Furthermore, the enhanced alkyl chain ordering and the membrane deformation energy induced by cholesterol increase the permeation free energy penalty. In contrast, cholesterol has minor effects on the water local diffusivities within IPA membranes. Overall, the cholesterol reduces the water permittivity of rigid IPA membranes due to the synergistic effects of increased alkyl chain ordering and enhanced membrane mechanical modulus. The results provide molecular insights into the effects of molecular packing and mechanical deformations on the water permittivity of biomimetic IPA membranes, which is critical for designing IPA vesicular carriers.

## 1. Introduction

Phospholipid is an amphiphilic biomolecule and the major component of cell membranes. Phospholipids can self-assemble in vitro into lipid vesicles, also termed liposomes, which can carry both hydrophilic and hydrophobic substances within the hydrophilic core and the bilayer shell, respectively [1]. Liposomes have been widely applied in pharmaceutics to modify drug adsorption, prolong drug biological half-life, and reduce drug toxicity and metabolism [2,3,4]. Other than phospholipids, studies have demonstrated the formation of vesicles with various amphiphiles, including ionic or non-ionic surfactants, and polymers [5]. Kaler et al. first demonstrated the spontaneous vesicle formation from the mixture of hexadecyltrimethylammonium tosylate and sodium dodecylbenzene sulfonate, and the resulting vesicles are termed “catanionic vesicles” [6]. Further removing residual counter ions from a 1:1 molar mixture of cationic/anionic amphiphiles results in a molecular complex termed ion pair amphiphile, IPA, where the cationic and anionic head groups are held together via electrostatic attraction. A dicatenar IPA complex composed of a pair of single-chain ionic amphiphiles is therefore a pseudo-double-tailed bio-mimic to a zwitterionic phospholipid, e.g., phosphatidylcholine and phosphatidylethanolamine. As liposome substitutes, IPA vesicles have great potentials in cosmetics, transdermal delivery, and pharmaceutical applications [7,8].

Compared with liposomes, IPA vesicles have the advantage of high chemical stability against hydrolysis. Yet, IPA vesicles in general have lower physical and colloidal stability. Common strategies to improve the colloidal stability of catanionic vesicles include inter-vesicular and intra-vesicular modifications [7]. The inter-vesicular repulsion between catanionic vesicles, for instance, can be achieved by introducing additional charged double-tailed amphiphile into the dicatenar IPA vesicles [9,10,11]. In addition, the electrostatic repulsion therefore prohibits the vesicle collisions, improving the long term colloidal stability. The intra-vesicular modifications can be accomplished via introducing stabilizing additives such as cholesterol that strengthen the stability and the mechanical properties of the IPA vesicular bilayer [12,13,14]. Cholesterol is a common biological membrane additive known to alter the mechanical properties and fluidity of the lipid membranes. Previous simulation study by Kuo et al. demonstrated that cholesterol stabilizes alkyltrimethylammonium-alkylsulfate IPA bilayers in a similar manner as lipid bilayers [13]. Our preliminary simulation study on the same IPA membrane systems mixed with cholesterol further illustrated that cholesterol preferential interacts with anionic alkylsulfate, increasing the contribution of anionic component to the overall mechanical modulus [14,15].

At a given temperature, a lipid bilayer can exist in either gel (solid, S) or fluidic (or liquid disordered, Ld) phase. The S phase is a solid-like phase where most alkyl chains are aligned within the hydrophobic region of the bilayer; while in the Ld phase, lipid molecules have more disordered hydrophobic chains and can diffuse freely in lateral dimensions. For a Ld phase phospholipid bilayer, adding cholesterol can induce the ordering of neighboring alkyl chains by the rigid sterol ring, leading to an increased local membrane rigidity [16]. In contrast, adding cholesterol into a S phase lipid bilayer disrupt the packing of nearby hydrocarbon chains. Biomimetic IPA bilayers exhibit similar phase properties to the phospholipid bilayer systems [17,18]. Also, the cholesterol additives give rise to similar effects on the IPA bilayer’s phase behavior and mechanical properties as the phospholipid bilayers [19]. Recent studies showed that high cholesterol content can stabilize the IPA vesicles, possibly due to the vanishing of the local phase separation [20]. These results demonstrate a close structural correlation and similar response to cholesterol between the biomimetic IPA and the lipid bilayer system.

A nonspecific diffusion for the substance permeating across the bilayer, also denoted as the passive membrane transport, is driven by the concentration gradient [21]. Theoretically, the permeation of a substance is characterized by the permeation coefficient *P*, which is related to the molecular flux *J* across a bilayer and the concentration gradient Δc [22,23]:

(1) $$\begin{array}{c} P=\frac{J}{\Delta c}. \end{array}$$

A common permeation model for a lipid bilayer is the inhomogeneous solubility-diffusivity model, in which the permeability across a membrane with the thickness *h* is expressed as [23,24]:

(2) $$\begin{array}{c} \frac{1}{P}=\int_{0}^{h}\frac{1}{K(z)D(z)}dz \end{array}$$

where K(z) and D(z) are the position-dependent partition coefficient and diffusion coefficient of the target substance. The model combined with computer simulation have been applied to study the rates and molecular mechanisms of the permeation of various molecules across bilayers [21,25,26,27,28]. The factors affecting the membrane permittivity include the composing lipids, the corresponding phases, and the packing characteristics, etc. In general, S phase lipid bilayers exhibit higher mechanical strength and lower membrane permittivity. Also, liposomes composed of saturated lipids with cholesterol exhibits lower membrane permittivity. Recent studies further showed that the fluctuations in the membrane conformation and the potential energy are critical to the permeation of hydrophilic molecules [29].

Passive transport is the mechanism for most of small neutral molecules and drug molecules. Hence, it is important to understand the process of passive permeation for medical and pharmaceutical applications. In this work, we applied molecular dynamics (MD) simulations to characterize the water permittivity of the biomimetic IPA membrane. Our early studies demonstrated the molecular effect of cholesterol on modulating the structural and mechanical properties of IPA membranes [14]. Here, we further examined on the mechanisms of cholesterol on modulating the IPA membrane permittivity form the structural, thermodynamic, and kinetic perspectives. The combined results provides important insights into IPA vesicle leakage stability and the membrane permeation of hydrophilic substance.

## 2. Results and Discussion

**Figure 1:**
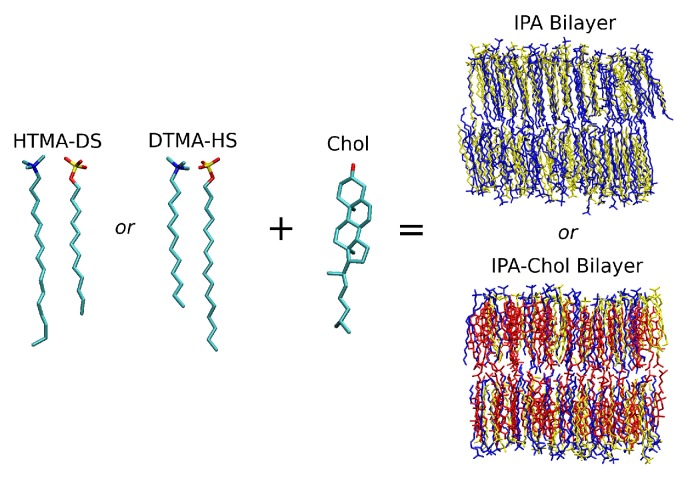
Molecular structures of HTMA-DS, DTMA-HS IPA complexes, and cholesterol. The representative bilayer structures of pure IPA and IPA-Chol systems are also shown, where the molecule color codes are: alkyltrimethylammonium in blue, alkylsulfate in yellow, and cholesterol in red. Graphics were generated using VMD package [30].

The target IPA series were the hexadecyltrimethylammonium-dodecylsulfate (HTMA-DS, CH${}_{3}$(CH${}_{2}$)${}_{15}$N(CH${}_{3}$)${}_{3}^{+}$-CH${}_{3}$(CH${}_{2}$)${}_{11}$SO${}_{4}^{-}$) and dodecyltrimethylammonium-hexadecylsulfate (DTMA- HS, CH${}_{3}$(CH${}_{2}$)${}_{11}$N(CH${}_{3}$)${}_{3}^{+}$-CH${}_{3}$(CH${}_{2}$)${}_{15}$SO${}_{4}^{-}$), which have inverse alkyl chain asymmetry as illustrated in Figure 1. All molecular dynamics (MD) simulations were conducted under the isothermal-isobaric condition at 1 bar and 298 K. According to early experimental and simulation works, both systems are in S phase at 298 K [15,17]. Different cholesterol (Chol) concentrations were introduced into IPA bilayers to investigate the molecular effects of cholesterol on modulating the water permittivity of the biomimetic IPA membrane. The molecular compositions for the two IPA-Chol bilayer systems utilized in the presented MD studies are listed in Table 1.

### 2.1. Bilayer Structural and Mechanical Properties

It is known that cholesterol can alter the structural and mechanical properties of lipid and IPA bilayers. To characterize the effect of cholesterol on the S phase HTMA-DS and DTMA-HS IPA bilayer structures, we first analyzed the alkyl chain conformation and ordering via deuterium order parameter ($|S_{\mathrm{CD}}|$) and gauche conformer fraction. In MD simulation, the deuterium order parameter, $|S_{\mathrm{CD}}|$, can be evaluated as: [31,32]

(3) $$S_{\mathrm{CD}}=\frac{1}{2}\langle 3cos^{2}\left(\theta \right)-1\rangle ,$$

where θ denotes the angle between the C-H bond and the bilayer normal, and the angle brackets represent the ensemble average. According to the study by Chen et al., a Ld phase IPA bilayer has the $|S_{\mathrm{CD}}|$ profile smaller than 0.3, and a S phase lipid bilayer has the one larger than 0.3 [15]. Meanwhile, the gauche fraction is evaluated as the fraction of gauche conformers along alkyl chains, where a gauche conformer is defined for the alkyl dihedral angle between −120 and 120 degrees. Smaller gauche fraction values indicate the higher chain ordering with more extended alkyl chains [33]. Chen et al. also determined the threshold gauche faction of 0.15 to distinguish Ld and S phase [15].

**Figure 2:**
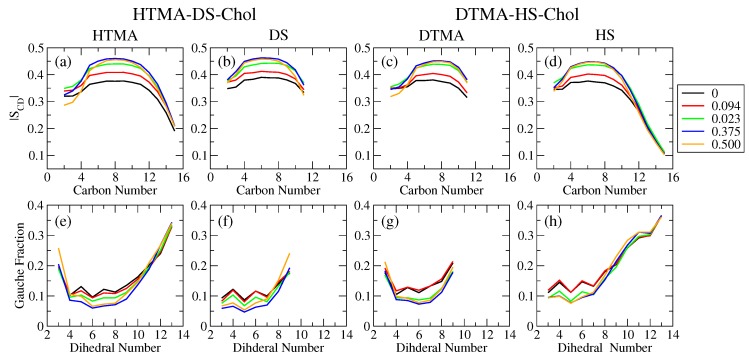
The deuterium order parameter ($|S_{\mathrm{CD}}|$) profiles at various X${}_{\mathrm{chol}}$ of (**a**) HTMA and (**b**) DS components for HTMA-DS-Chol systems and (**c**) DTMA and (**d**) HS components for DTMA-HS-Chol systems are shown in top panels. Also, the gauche fraction profiles at various X${}_{\mathrm{chol}}$ along the alkyl chains of (**e**) HTMA and (**f**) DS components for HTMA-DS-Chol systems and (**g**) DTMA and (**h**) HS components for DTMA-HS-Chol systems are shown in bottom panels.

For both pure IPA bilayer systems, i.e., HTMA-DS and DTMA-HS systems, the $|S_{\mathrm{CD}}|$ profiles have the plateau values of 0.45 and the gauche fraction profiles have the plateau values below 0.15 as illustrated in Figure 2. These results confirm that both pure HTMA-DS and DTMA-HS IPA bilayers are in S phase at 298 K. Upon the addition of Chol, the $|S_{\mathrm{CD}}|$ values increase and the gauche fractions decrease with X${}_{\mathrm{chol}}$ for the middle alkyl segments of all the surfactant species in both IPA systems. This suggests that the chain order is enhanced by the rigid sterol ring of cholesterol. Furthermore, the HTMA-DS-Chol system has slightly higher $|S_{\mathrm{CD}}|$ and lower gauche fraction than the DTMA-HS-Chol system, particularly near the terminus of alkyl chains. This indicates that the HTMA-DS bilayer have higher alkyl chain ordering than the DTMA-HS system, due to the intrinsic alkyl chain mismatch between the alkyltrimethylammonium and alkylsulfate [15].

**Figure 3:**
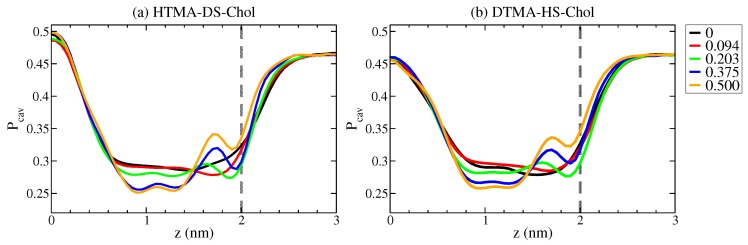
Cavity density profile $P_{\mathrm{cav}}\left(z\right)$ of (**a**) HTMA-DS-Chol and (**b**) DTMA-HS-Chol bilayers at various X${}_{\mathrm{chol}}$ with the standard deviations of less than 0.015. Error bars are not shown for clarity.

Several experimental and simulation studies showed that cholesterol can alter the molecular packing within a lipid membrane and modulate the water permeability of the membrane [34,35]. Here, we calculated the cavity density $P_{\mathrm{cav}}$ along the bilayer normal to probe the effects of cholesterol on the alkyl chain packing for HTMA-DS and DTMA-HS IPA membranes. The cavity density profile $P_{\mathrm{cav}}$ was evaluated using a uniform grid of the 0.5 Å gridsize [34]. Each bin was examined for any atom occupation to calculated the probability density at position z. As shown in Figure 3, the cavity density profiles for pure IPA systems peak at the bilayer center and is reduced to near 0.3 at z = 0.6–1.8 nm, similar to that for the lipid bilayers [34]. The low $P_{\mathrm{cav}}$ region for the HTMA-DS system is wider than the DTMA-HS membrane, which can be correlated with the higher alkyl chain ordering within the HTMA-DS bilayer. Further adding cholesterol reduces the $P_{\mathrm{cav}}$ values in the range of z = 0.6–1.4 nm, where the sterol ring are populated. In contrast, the $P_{\mathrm{cav}}$ in the region of z = 1.4–2.0 nm increases with the cholesterol concentration. These effects of cholesterol on the $P_{\mathrm{cav}}$ values become increasingly significant starting from X${}_{\mathrm{chol}}$ = 0.203. This can be correlated with the variations on the alkyl chain ordering as shown by the $|S_{\mathrm{CD}}|$ and gauche fraction analyses in Figure 2. The reduction of $P_{\mathrm{cav}}$ around the alkyl middle segments indicates a induced molecular packing by cholesterol which lowering the nearby free cavity. Similar effects have been reported on DPPC-Chol and sphingolipid membranes [34]. Yet, the increased $P_{\mathrm{cav}}$ near the hydrophilic region for IPA-Chol systems differ from the those reported for lipid membranes, in which cholesterol also reduces the $P_{\mathrm{cav}}$ near the hydrophilic region [34]. The reported lipid-cholesterol membrane studies were focusing on the lipid systems in Ld phase, compared with the S phase IPA bilayers in this work. According to previous studies on IPA systems, adding cholesterol into S phase IPA bilayers can increase the spacing between IPAs [13,14]. Thus, adding cholesterol disrupts the molecular packing within the hydrophilic region for both HTMA-DS and DTMA-HS bilayers, leading to increased cavity probability near the membrane surface.

**Figure 4:**
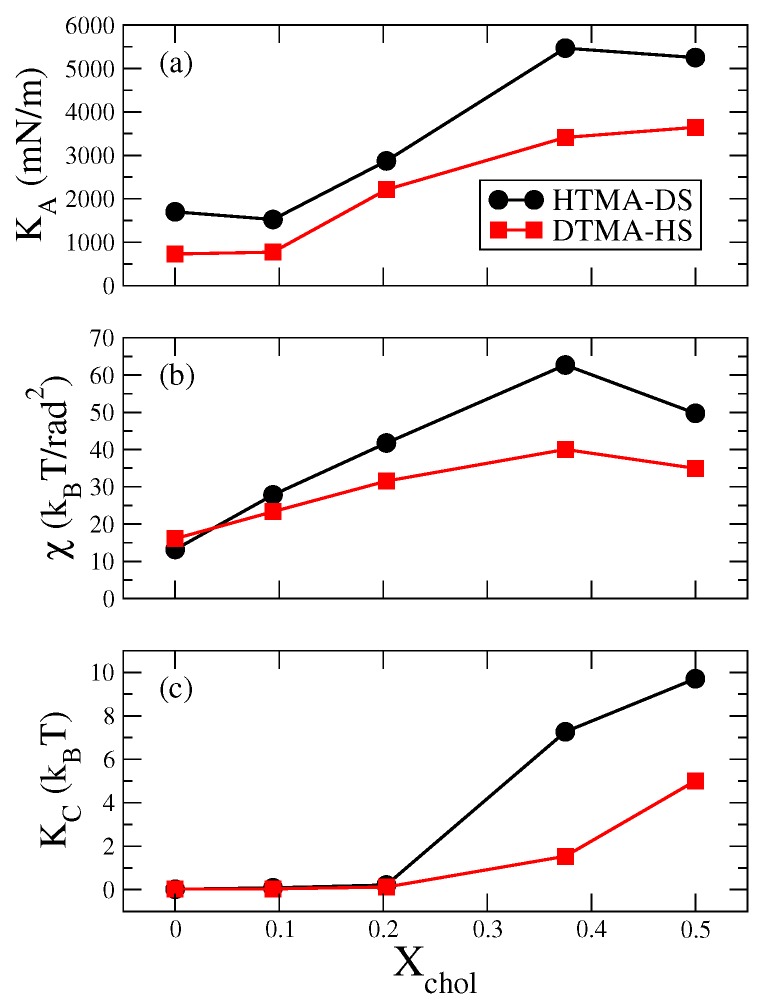
Three mechanical moduli for HTMA-DS-Chol (black circle) and DTMA-HS-Chol (red square) bilayers at various X${}_{\mathrm{chol}}$: (**a**) the area compressibility modulus $K_{A}$, (**b**) molecular tilt modulus χ, and (**c**) bending modulus $K_{C}$.

To characterize the effects of cholesterol on the mechanical properties of the IPA bilayers, we calculated the area compressibility modulus $K_{A}$, the molecular tilt modulus χ, and the bending modulus $K_{C}$. For the pure IPA bilayers, as shown in Figure 4, all three moduli ($K_{A}$, χ, and $K_{C}$) for HTMA-DS system are greater than those for the DTMA-HS system. This is attributed to the higher alkyl chain ordering within in the HTMA-DS system, as illustrated by the $|S_{\mathrm{CD}}|$ and gauche fraction analyses in Figure 2. The $K_{A}$ and χ values for both pure IPA systems are higher than the reported threshold values of $K_{A}=700$ mN/m and χ=13$k_{B}T$/rad${}^{2}$, respectively [15]. This also suggests that both HTMA-DS and DTMA-HS are in S phase at 298 K. After introducing cholesterol, the mechanical moduli for both IPA membranes increases. Such mechanical enhancement can be corresponded to the enhanced ordering of the middle alkyl chain segment induced by the rigid sterol ring of cholesterol. Around X${}_{\mathrm{chol}}$ = 0.375, both $K_{A}$ and χ are at their maxima while $K_{C}$ dramatically increase for both IPA-Chol systems. This is due to the competing effects among the induced ordering at the middle alkyl segment, the disordering at the alkyl tails, and increased spacing within the hydrophilic regions induced by cholesterol addition, consistent with the results reported by Huang et al. [14]. Furthermore, the DTMA-HS-Chol bilayers generally have lower mechanical strength than the HTMA-DS-Chol systems. This can be attributed to the more disordered alkyl tail region within the DTMA-HS-Chol membranes.

### 2.2. Free Energy of Water Crossing IPA-Chol Bilayer

**Figure 5:**
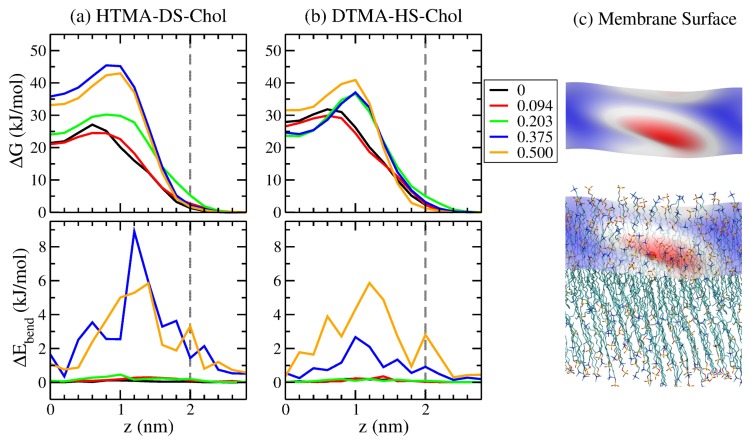
The free energy profiles (top panels) and the membrane bending energy profiles during water permeation for (**a**) HTMA-DS-Chol and (**b**) DTMA-HS-Chol bilayers at various X${}_{\mathrm{chol}}$. In addition (**c**) the representative membrane surface with maximum bending energy at z = 1.2 nm, and the superimposition of the surface with the bilayer structure where the permeant water oxygen is labeled with red sphere. Graphics were generated using VMD package [30].

According to the inhomogeneous solubility-diffusivity model as described in Equation (2), the water permittivity of the membrane depends on both the partition coefficient K(z) and the diffusion coefficient D(z) of the permeate water in the membrane [23,24]. The position-dependent partition coefficient K(z) can be related to the free energy of permeation ΔG(z) as:

(4) $$\begin{array}{c} K\left(z\right)=e^{-\Delta G\left(z\right)/k_{B}T}. \end{array}$$

where $k_{B}$ and *T* denote the Boltzmann constant and the temperature, respectively. In this work, we calculated the free energy profiles for a water molecule across the HTMA-DS-Chol and DTMA-HS-Chol bilayer systems, as shown in Figure 5. As the permeate water goes from the bulk phase toward the membrane interior of both pure HTMA-DS and DTMA-HS bilayers, the free energy starts raising at around 2.2 nm, close to the average positions of the IPA head groups at 2 nm. The free energy keeps increasing as the water moves toward the center of the bilayer, which can be attributed to the membrane hydrophobic region. Please note that there exhibits a local free energy minimum near the membrane center. Such free energy local minimum is a common feature for lipid bilayers in S phase [28,36,37], compared with the free energy barrier with a narrow plateau near the core observed for lipid bilayers in Ld phase [34,38]. This again supports that both IPA membranes are in S phase at 298 K. The local free energy minimum observed for IPA bilayers is resulted from the void near the bilayer center as shown in Figure 3, which is originated from the alkyl chain mismatch between two IPA components [13,14]. The void space with locally lowered density provides the penetrating water molecule a relatively stable region in the membrane hydrophobic region. For pure IPA bilayers, a larger mismatch near the membrane core is observed in DTMA-HS bilayer [15]. As illustrated by the lower cavity density for the DTMA-HS system in Figure 3, the voids near the bilayer center are hence filled with less ordered DTMA-HS alkyl tails. This results in a wider and higher basin around the free energy minimum for the DTMA-HS system.

With low amount of cholesterol addition (X${}_{\mathrm{chol}}$ = 0.094), the free energy profiles for water permeation for both IPA systems are only slightly affected where the locations of the maxima remain but the barrier heights reduces 1–2 kJ/mol. When adding a few cholesterol into the S phase IPA membranes, the cholesterol only marginally increases the alkyl chain ordering shown in Figure 2. Meanwhile, low amount of cholesterol slightly increase the cavity density in the region around z = 1 nm, which corresponds to the minor reduction of free energy barriers for both HTMA-DS-Chol and DTMA-HS-Chol systems. With the cholesterol addition of X${}_{\mathrm{chol}}>0.203$, the water permeation free energy for both IPA bilayers increases. This is attributed to the changes in the cavity density starting at X${}_{\mathrm{chol}}$ = 0.203. The increased barriers observed for both IPA-Chol system at z = 1 nm can be related to the reduction cavity in the region of z = 0.6–1.4 nm induced by the ordering effect of the cholesterol sterol ring. Comparing the two IPA systems, both IPA systems have the permeation barrier of around 30 kJ/mol for X${}_{\mathrm{chol}}\leq 0.203$. Further increasing X${}_{\mathrm{chol}}>0.375$, the HTMA-DS-Chol bilayers exhibits larger free energy barrier than the DTMA-HS-Chol bilayers, which is partly attributed to the more ordered structure in the HTMA-DS-Chol systems.

As the permeate water entering the membrane, it can cause local membrane deformation as demonstrated by the representative simulation snapshot in Figure 5. Such membrane deformation indicates that the membrane mechanical energy also affects the water permeation across a S phase IPA membrane. To characterize the contribution of the membrane deformation to the overall free energy, we evaluated the bending energy $\Delta E_{\mathrm{bend}}$ using the Helfrich free energy via integration over the membrane surface [39]:

(5) $$\begin{array}{c} \Delta E_{\mathrm{bend}}=\int \frac{K_{C}}{2}{\left(c_{1}+c_{2}\right)}^{2}\;dA, \end{array}$$

where $K_{C}$ is the bending modulus. The parameters $c_{1}$ and $c_{2}$ denote the two principal curvatures, and can be obtained from the membrane height field h(x,y) defined as the positions of the bilayer headgroups in the normal direction [40]. Here, the membrane height field h(x,y) was approximated as:

(6) $$\begin{array}{c} h\left(x,y\right)=\sum_{m=0}^{2}\sum_{n=0}^{2}\left(a_{m,n}sin\left(\frac{2m\pi }{L_{x}}+\frac{2n\pi }{L_{y}}\right)+b_{m,n}cos\left(\frac{2m\pi }{L_{x}}+\frac{2n\pi }{L_{y}}\right)\right), \end{array}$$

where ($L_{x}$, $L_{y}$) and (*m*, *n*) denote the system box size and the wave vectors in x and y dimensions, respectively; and $a_{m,n}$ and $b_{m,n}$ are the fitting parameters for the surface h(x,y). The analytical expression of h(x,y) thus allowed us to determine $c_{1}$ and $c_{2}$ for any (x,y) coordinate on the membrane surface.

As illustrated in Figure 5, the membrane bending energy profile for water permeation have the maximum at z = 1.2 nm, close to the free energy barrier at z = 1 nm. This suggests that the membrane deformation also provides additional water permeation barriers of the IPA membrane. As the X${}_{\mathrm{chol}}$ increases, the bending energy barrier also raises due to the enhanced bending modulus $K_{C}$ as shown in Figure 4. However, the HTMA-DS-Chol system with X${}_{\mathrm{chol}}=0.375$, despite of its smaller $K_{C}$, has a higher bending energy than the X${}_{\mathrm{chol}}=0.5$ system. This is because the high $K_{C}$ for the X${}_{\mathrm{chol}}=0.5$ reduces the deformation of the membrane, resulting in smaller surface curvatures and hence a lower bending energy barrier. Please note that in the region of z = 1.4–2.0 nm, the $P_{\mathrm{cav}}$ increases with X${}_{\mathrm{chol}}$, which should lead to decreased free energy. Yet, the variation of $\Delta E_{\mathrm{bend}}$ is more dominate over the cavity effect when adding cholesterol, leading to overall increased free energy in the hydrophilic region. According to our early study on comparing IPA and phospholipid bilayers, IPA membranes have higher mechanical strength than lipid systems [18]. Hence, the effects of membrane deformation should be considered for the water permeation across most types of IPA bilayers. Comparing the two IPA-Chol systems, the membrane bending energy for HTMA-DS-Chol systems are greater than that for DTMA-HS-Chol systems when X${}_{\mathrm{chol}}>0.375$, attributed to the higher $K_{C}$ of the HTMA-DS-Chol systems. This also contributes to a higher permeation free energy of HTMA-DS-Chol membranes with high cholesterol content.

### 2.3. Water Permittivity of IPA-Chol Bilayer

**Figure 6:**
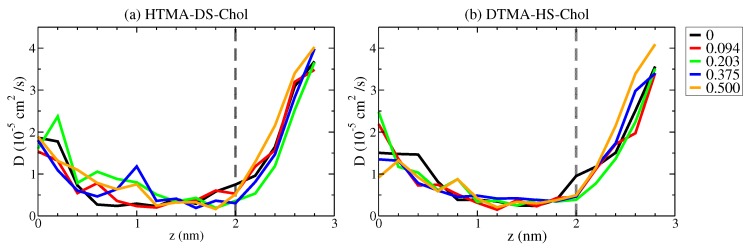
The local diffusivity profiles D(z) of the permeant water for (**a**) HTMA-DS-Chol and (**b**) DTMA-HS-Chol bilayers at various X${}_{\mathrm{chol}}$.

Other than the permeation free energy ΔG(z), the position- dependent diffusion coefficient D(z) is also an important parameter to evaluate water permittivity of IPA-Chol Bilayer. Here, we calculated the local diffusion coefficient using the force autocorrelation function [21]:

(7) $$\begin{array}{c} D\left(z\right)=\frac{{\left(RT\right)}^{2}}{\int_{0}^{\infty }\langle \Delta F_{z}\left(t\right)\Delta F_{z}\left(0\right)\rangle dt} \end{array}$$

where *R* is the gas constant and $\Delta F_{z}\left(t\right)=F_{z}\left(t\right)-\langle F_{z}\rangle$ is the instantaneous force deviation from the mean force along the bilayer normal. As shown in Figure 6, the local diffusivity profiles for all IPA-Chol bilayers show plateau of lower D(z) at around 1.4 to 2 nm. In this region, water molecule experiences both the hydrophobic repulsion from the carbon segments and the hydrophilic association from the polar groups, leading to slower dynamics, similar to that observed in the lipid systems [34]. In addition, the alkyl chain ordering altered by cholesterol addition does not significantly affect the local diffusivity profile near the hydrophobic region. As the permeate water entering the bilayer, the membrane deformation can decreases the nearby alkyl chain ordering, leading to increased local diffusivity. Such effects can be observed for HTMA-DS-Chol system in the range of z = 0.6–1.2 nm: the packing disruption induced by deformation enhances with increased X${}_{\mathrm{chol}}$, resulting in increased local diffusivity in the region. However, compared with the large dependency of the free energy profile on the cholesterol concentration, the local diffusivity profiles exhibit less obvious changes upon varying X${}_{\mathrm{chol}}$.

With the results of free energy profiles and local diffusivity profiles shown in Figure 5 and Figure 6, respectively, the local permeation resistance $R_{local}\left(z\right)$ can be calculated as [23]:

(8) $$\begin{array}{c} R_{local}\left(z\right)=\frac{1}{K(z)D(z)}=\frac{e^{\Delta G\left(z\right)/k_{B}T}}{D(z)}. \end{array}$$

**Figure 7:**
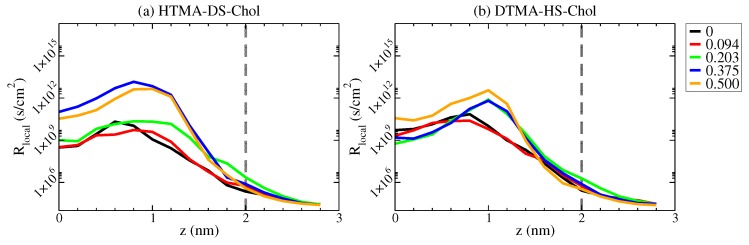
The local permeation resistance profiles $R_{local}\left(z\right)$ for (**a**) HTMA-DS-Chol and (**b**) DTMA-HS-Chol bilayers at various X${}_{\mathrm{chol}}$.

Figure 7 shows the local permeation resistance profile for the HTMA-DS-Chol and DTMA-HS-Chol systems. Comparing the free energy profiles and local diffusivity profiles, the overall variation in local diffusivity has little dependence on X${}_{\mathrm{chol}}$. Hence, the local resistance difference depends primarily on the variance in the free energy of permeation. For all the systems containing cholesterol, the region between 0.6 to 1.4 nm shows higher local resistance than other regions, corresponding to where cholesterol sterol rings reside. This suggests that the enhanced alkyl chain ordering and reduced cavity density induced by cholesterol addition decreases the water local permeation. In addition, with cholesterol addition, the increased permeation free energy induced by the enhanced mechanical modulus leads to the increased $R_{local}\left(z\right)$ in the hydrophilic region of z =1.4−2.0 nm.

Integrating the values of local resistance along the permeation path yields the overall permeation resistance, or the reciprocal of permeability:

(9) $$\begin{array}{c} R=\frac{1}{P}=\int R_{local}\left(z\right)=\int \frac{e^{\Delta G\left(z\right)/k_{B}T}}{D(z)} \end{array}$$

**Figure 8:**
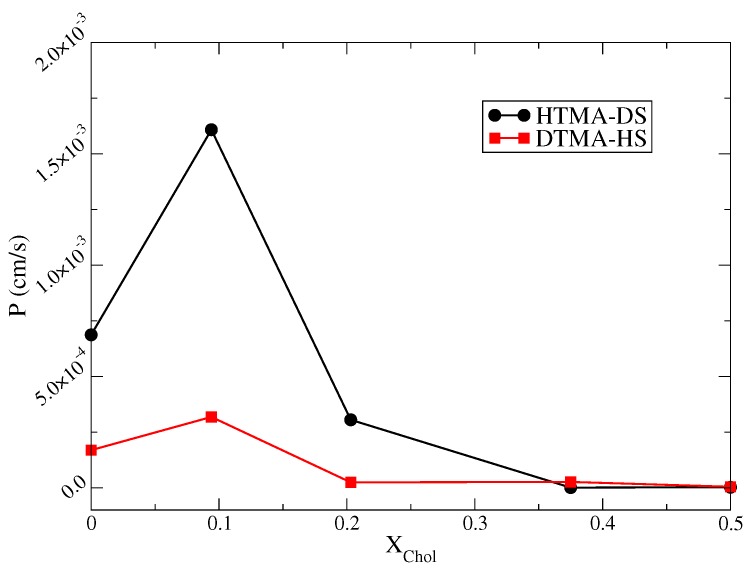
The water permittivity coefficients for HTMA-DS-Chol (black circle) and DTMA-HS-Chol (red square) bilayers as a function of cholesterol concentration X${}_{\mathrm{chol}}$.

Figure 8 shows the resulting permeabilities for HTMA-DS-Chol and DTMA-HS-Chol bilayers. Compared with two IPA-Chol system, the DTMA-HS-Chol membrane has lower permeability, resulting form the lower cavity density near the bilayer center where the voids between two leaflet filled with less ordered alkyl tails. Since $R_{local}\left(z\right)$ primarily depends on the permeation free energy, the variations in permeability can be mainly interpreted by the free energy changes induced by cholesterol. For both types of IPA-Chol systems, the permeabilities increase at X${}_{\mathrm{chol}}$ = 0.094 due to the slightly decreased free energy barrier. When further increasing X${}_{\mathrm{chol}}$, the permeabilities dramatically decrease 1-2 orders of magnitudes. Above X${}_{\mathrm{chol}}$ = 0.375, the permeabilities for both IPA-Chol membranes become similar and decline to $2.3^{-6}$ cm/s for HTMA-DS-Chol and $4.6\times 10^{-6}$ cm/s for DTMA-HS-Chol at X${}_{\mathrm{chol}}$ = 0.5. Such permeability reduction results from the synergistic effects of induced alkyl chain ordering and the enhanced membrane bending mechanics upon cholesterol addition. Please note that adding cholesterol into phospholipid bilayers may induced local phase seperation, resulting in a complex permittivity response [28]. Yet, no local phase domains were observed in this work, possibly due to the relatively small system size. In addition, the phase domain effect on the IPA membrane permittivity will be examined in the future.

## 3. Materials and Methods

### 3.1. Simulations Details

All molecular dynamics (MD) simulations were conducted with Gromacs 5.0.4 package with periodic boundary conditions applied in all three dimensions [41,42]. Each system was composed of 128 IPA and cholesterol with 64 molecules per leaflet and fully hydrated with 3464 water molecules. The initial configurations for the HTMA-DS-Chol and the DTMA-HS-Chol bilayers were constructed via Packmol [43]. All simulations were performed using the isothermal-isobaric (NPT) ensemble. Temperature and pressure were maintained at 298 K and 1 bar using the Nos$\overset{´}{e}$-Hoover and semi-isotropic Parrinello-Rahman algorithm, respectively [44,45,46,47]. The Lennard-Jones and short-range electrostatic potentials were cut off at 1.2 nm where the Lennard-Jones interactions were smoothly shifted to zero starting from 0.8 nm. Long-range electrostatic interactions were evaluated using Particle mesh Ewald (PME) method [48,49]. All bonds were constrained at their equilibrium length using the LINCS algorithm [50]. A 2 fs integration timestep was used to evaluate the equations of motions of atoms.

The CHARMM36 united-atom (C36-UA) force field parameters were applied for IPA and cholesterol molecules and the TIP3P model for water [51,52,53]. This force field combination have been used in various MD studies on alkyltrimethylammonium-alkylsulfate iPA and phospholipid bilayers [15,51]. Each bilayer system was first energy minimized via the steepest descent minimization algorithm, then equilibrated at 348 K and 1 bar for 40 ns to ensure the bilayer in Ld phase. The IPA bilayer system was then annealed from 348 K to 298 K with a 2.5 K/ns cooling rate, allowing the bilayer to naturally transition to S phase. After the annealing process, each simulation was first equilibrated at 298 K and 1 bar for 40 ns followed by a production run of 160 ns, in which system configurations were saved every 10 ps for analyses of the membrane structural and mechanical characteristics. All analyses were conduced with the in-house codes following the algorithms described in the main text.

### 3.2. Mechanical Modulus

To characterize the mechanical properties of the IPA-Chol bilayers, we calculated three different mechanical moduli, including the area compressibility modulus $K_{A}$, the molecular tilt modulus χ, and the bending modulus $K_{C}$. The area compressibility modulus $K_{A}$ characterizes the bilayer resistance against the membrane lateral deformation and can be calculated from MD trajectories as [54,55]:

(10) $$\begin{array}{c} K_{A}=\frac{k_{B}T\langle A_{mol}\rangle }{N\langle \delta A_{mol}^{2}\rangle }\;, \end{array}$$

where $k_{B}$ is the Boltzmann constant, *T* is the simulated temperature, $\langle A_{mol}\rangle$ is the average lateral area per molecule, $\langle \delta A_{mol}^{2}\rangle$ is the variance of $A_{mol}$, and N=64 denotes the number of the molecule per leaflet in our simulation. Meanwhile, the molecular tilt modulus, χ, characterizes the resistance against the alkyl chain tilting within the bilayer, and can be obtained via the quadratic fitting to the free energy profile of molecular tilting F(α) [56,57]. In addition, the tilting free energy profile F(α) was evaluated from the Boltzmann inversion of the normalized tilt angle distribution P(α):

(11) $$\begin{array}{c} F\left(\alpha \right)=-k_{B}Tln\left[\frac{P(\alpha )}{\sin(\alpha )}\right]=F\left(\alpha_{0}\right)+\frac{\chi }{2}{\left(\alpha -\alpha_{0}\right)}^{2}\;, \end{array}$$

where α denotes the molecular tilt of the alkyl chain, sin(α) is the normalizing Jacobian factor, and $F(\alpha_{0})$ is the free energy minimum at the equilibrium tilt angle $\alpha_{0}$. Here, α was defined as the angle between bilayer normal and the alkyl chain direction vector, i.e., the vector connecting the first and the second last carbons of the alkyl chain. Lastly, the bilayer bending modulus $K_{C}$ characterizes the energetic costs of the membrane bending deformations. Here, we extracted $K_{C}$ of IPA-Chol bilayers from MD simulations using the spectrum approach based on the modified Helfrich-Canham theory [40]. The bilayer deformation is quantified during the simulation via the height field h(x,y) defined as the positions of the bilayer hydrophilic groups in the normal direction. After Fourier transform, the power spectrum of the height fluctuations is predicted as a function of the wavefactor *q* [58]:

(12) $$\begin{array}{c} {\langle |h\left(q\right)|}^{2}\rangle =\frac{k_{B}T}{K_{C}q^{4}}+\frac{k_{B}T}{\chi q^{2}}\;, \end{array}$$

which includes the contributions of membrane bending and lipid tilting to the overall deformations.

### 3.3. Permeation Free Energy Calculation

The permeation free energy profiles of water across IPA-Chol bilayers were evaluated with the constrained molecular dynamics as a function of the *z* distance between the permeant water and the membrane center of mass, *z* [59]. Each simulation was conducted by constraining at a fixed *z* value with a stiff harmonic spring constant of 10${}^{5}$ kJ/mol/nm${}^{2}$. A total of 15 constraint values were chosen every 0.2 nm in the range of 0 to 2.8 nm. For each *z* value, 10 different initial configurations were used to obtain the sufficient ensemble sampling. The 10 configurations were extracted every 1 to 10 ns from a 100 ns trajectory of NPT equilibrium simulation. In each extracted configuration, one water molecule was inserted randomly into free cavity at a desired *z* location. Then, an energy minimization was applied to eliminate the bad contacts between the inserted water and the surrounded atoms. With these 15 times 10 configurations, constrained MD runs were carried out for 500 ps each at fixed *z* locations under the canonical (NVT) ensemble to record the mean constraint forces 〈f(z)〉. The permeation free energy, ΔG(z) was then obtained from the mean constraint forces via thermodynamic integration:

(13) $$\begin{array}{c} \Delta G\left(z\right)=\int_{0}^{2.8\;\mathrm{nm}}\langle f\left(z\right)\rangle dz. \end{array}$$

Please note that the instantaneous constraint forces f(z) were also used to calculate local diffusivity D(z) via Equation (7).

## 4. Conclusions

An all-atom molecular dynamics simulation was applied to examine how cholesterol addition with X${}_{\mathrm{chol}}$ = 0–0.5 affects the structural and permeation properties of the S phase biomimetic bilayers composed of hexadecyltrimethylammonium-dodecylsulfate (HTMA-DS) and dodecyltrimethylammonium-hexadecylsulfate (DTMA-HS). Simulation results showed that DTMA-HS-Chol systems have an overall smaller degree of chain ordering compared with HTMA-DS-Chol systems because of the greater intrinsic alkyl chain mismatch near the core region [14,15]. Cholesterol addition also enhances the membrane mechanical properties for both HTMA-DS and DTMA-HS systems, where the HTMA-DS-Chol bilayers have higher mechanical strengths owing to the more ordered alkyl chain packing. The combined effects of molecular packing and mechanical modulation cause the water permeation free energy barrier to slightly decrease at X${}_{\mathrm{chol}}=0.094$ and to significantly increase when X${}_{\mathrm{chol}}\geq 0.203$ for both HTMA-DS and DTMA-HS systems. The analyses of membrane deformation energy further demonstrate that the enhanced mechanical strength induced by cholesterol can contribute additional energy costs for water permeations. In contrast, the local diffusivity is less affected by cholesterol addition.

Combining both free energy and local diffusivity data, we summarized the overall effects of cholesterol on the water permittivity of HTMA-DS and DTMA-HS membranes. With a low cholesterol amount of X${}_{\mathrm{chol}}=0.094$, the water permittivity for both IPA systems slightly increases due to the slight reduction of the free energy barrier. When X${}_{\mathrm{chol}}\geq 0.203$, the synergistic effects of increased alkyl ordering and enhanced mechanical strength leads to a dramatical reduction of water permittivity of both HTMA-DS-Chol and DTMA-HS-Chol bilayers. Please note that the main phase transition temperatures for IPA systems are higher than the corresponding lipid systems, suggesting that the biomimetic IPA membranes are in S phase with higher mechanical strength under the physiological condition. Hence, the modulation of molecular packing and mechanical properties becomes important for controlling the permittivity of IPA membranes. Other than cholesterol addition, chemical penetration enhancers such as ethanol may also be applied to modulate the IPA membrane permittivity. In addition, the mechanical and free energy analyses used in this work can provide invaluable molecular insights into the mechanisms of modulating the rigid biomimetic membrane permittivity for various delivery systems.

## Figures and Tables

**Table 1:** Compositions of bilayer systems comprising HTMA-DS or DTMA-HS IPA, cholesterol (Chol) and water with the mole fraction of cholesterol (X${}_{\mathrm{Chol}}$) ranged from 0 to 0.5.

System	X${}_{\mathrm{Chol}}$	N${}_{\mathrm{IPA}}$	N${}_{\mathrm{Chol}}$	N${}_{\mathrm{water}}$
	0	128	0	
HTMA-DS	0.094	116	12	
or	0.203	102	26	3464
DTMA-HS	0.375	80	48	
	0.5	64	64	

## Abbreviations

The following abbreviations are used in this manuscript:

IPA	Ion Pair Amphiphile
MD	molecular dynamics
HTMA	hexadecyltrimethylammonium
DTMA	dodecyltrimethylammonium
HS	hexadecylsulfate
DS	dodecylsulfate
Chol	cholesterol
